# Bioinformatic prediction of the AP2/ERF family genes in *Eucalyptus grandis*: focus on the CBF family

**DOI:** 10.1186/1753-6561-5-S7-P165

**Published:** 2011-09-13

**Authors:** Sahar Azar, Helène SanClemente, Gilles Marque, Christophe Dunand, Christiane Marque, Chantal Teulieres

**Affiliations:** 1 UMR5546 University of Toulouse, France

## Background

Due to their very high economic importance, Eucalyptus tree species are among the most planted hardwoods in the world with over 20 million hectares. However, as long-lived evergreen species, this genus is particularly exposed to cold. Frost tolerance varies among species and is inversely correlated to productivity. The AP2/ERF gene family includes developmentally and physiologically important transcription factors characterized by the presence of the AP2/ERF DNA-binding domain. AP2 proteins contain two AP2-like domains and RAV family proteins contain one AP2 domain and one B3 domain. ERF family proteins exhibit only one AP2 domain and are further divided into the DREB subfamily and the ERF subfamily [1]. The CBF/DREB1 protein differ from the other DREB proteins by the presence of “signature sequences” (PKK/RPAGRxKFxETRHP and DSAWR) flanking the DNA-binding AP2 domain [2]. The DREB factors recognize the C-repeat or dehydration response element (DRE) in the promoters of low temperature and/or water deficit responsive genes and would play a crucial role in response to abiotic stresses. CBF/DREB1 are the key regulators of the cold-responsive (COR) genes. So far CBF transcription factors have been mainly characterized in model plants such as Arabidopsis, but lately they were identified in several tree species including Eucalyptus [2]. The Eucalyptus cold tolerance was greatly improved in our hands when two genes from the four CBF members isolated from a tolerant species *E. gunnii* were individually constitutively overexpressed in the frost sensitive *E. urophylla* x *E. grandis* hybrid [3]. In the present study *E. grandis* AP2/ERF family genes were identified based on the presence of putative encoding AP2-domain(s) and were studied with regard to the model herbaceous Arabidopsis as well as the main sequenced woody plants. Within this family, a part of the study focused on the CBF/DREB1 subfamily which was compared to the four genes already characterized in *E. gunnii*[*2*].

## Methods

We used the available annotated *E. grandis* genome sequence at phytozome (http://www.phytozome.net/eucalyptus.php), the *Arabidopsis thaliana* AP2/ERF family downloaded from the DATF (Database of Arabidopsis Transcription Factors) database website (http://datf.cbi.pku.edu.cn/), and the sequences of AP2/ERF gene family from the grapevine, and the poplar obtained from several publications. In order to get the exhaustive gene AP2 family and the location of the genes of the *E. grandis* genome, we used Scipio [4]. Every identified protein was analyzed for structural motifs by scanning them against PROSITE patterns and profiles (http://www.expasy.org/prosite) and against Pfam (http://pfam.sanger.ac.uk/) to make sure of the presence of AP2 and/or the B3 domains. A fine correction of the predicted AP2/ERF proteins was performed. In order to identify all the members of CBF subfamily, a search within the *E. grandis* annotated AP2/ERF family was performed using consensus sequence including the two highly conserved signatures (PKKPAGR and DSAWR) surrounding the DNA-binding AP2-domain. As a check, we confirmed by blasting the sequences on the NCBI blast page. From a multiple alignment analysis performed with Clustal W, the phylogenetic trees were generated using MEGA version 5. The resulting phylogenetic trees were based on the maximum likelihood (ML) method [5].

## Results and main conclusions

Based on the sequence alignement, the phylogenetic analyses, and the known criteria described in litterature, this study revealed that the annotated AP2 genes from *E. grandis* were divided into the four subgroups already described for Arabidopsis (DREB, ERF subfamilies, AP2 subfamily and RAV subfamily). The two subfamilies DREB and ERF were separated according to the similarity of the sequences of the AP2/ERF domain and to the amino acid at 14 and 19 positions. Distribution of the DREB members in the four dicotyledonous plant genomes (Vitis, Arabidopsis, Populus and Eucalyptus) shows similarities between grapevine and *E. grandis* except for the A1 subgroup which corresponds to the CBF genes. Interestingly this group is much larger in *E. grandis* compared to the three other plant species. The 17 CBF genes (including one pseudogene) identified on *E. grandis* genome exhibit a very good conservation when compared to the *E. gunnii* CBF genes. The *E. grandis* CBF genes could be classified into the four groups (a, b, c et d) described for *E. gunnii* CBF. Most of the *Egr*CBF were localized on the chromosome 1, one member is on the chromosome 4 and the last two on the chromosome 5.
